# Pituitary adenoma apoplexy caused by rupture of an anterior communicating artery aneurysm: case report and literature review

**DOI:** 10.1186/s12957-015-0653-z

**Published:** 2015-07-30

**Authors:** Kan Xu, Yongjie Yuan, Jing Zhou, Jinlu Yu

**Affiliations:** Department of Neurosurgery, First Hospital of Jilin University, 71 Xinmin Avenue, Changchun, 130021 China

## Abstract

**Background:**

Pituitary adenoma combined with intracranial aneurysm is not rare. Some aneurysms are located inside pituitary adenomas, and most do not rupture. Pituitary apoplexy caused by aneurysm rupture is rare and is easily misdiagnosed as simple pituitary adenoma apoplexy.

**Case presentation:**

In this study, we report one case of rare pituitary adenoma apoplexy caused by the rupture of an anterior communicating artery aneurysm. The patient was a 49-year-old male who had an untreated pituitary adenoma for 3 years. The patient experienced a sudden headache; computed tomography (CT) and magnetic resonance imaging (MRI) revealed pituitary adenoma apoplexy and significant subarachnoid hemorrhage. Cranial CT angiography (CTA) showed a communicating artery aneurysm. Supratentorial intracranial aneurysm clipping and pituitary adenoma resection were performed. The aneurysm was a ruptured aneurysm located inside the pituitary adenoma. During the surgery, the aneurysm was clipped, and the majority of the tumor was resected. The patient recovered well after the surgery and received radiotherapy.

**Conclusions:**

This rare case demonstrates that when pituitary adenoma apoplexy is combined with subarachnoid hemorrhage, the possibility of a combined intrasellar aneurysm should be considered. During transsphenoidal tumor resection, aneurysm rupture should be avoided to prevent disastrous consequences.

## Background

The coexistence of a cerebral aneurysm and a pituitary adenoma is not uncommon; however, the presence of an aneurysm inside a pituitary adenoma is rare [1]. Among the cases of pituitary adenoma associated with aneurysm, most aneurysms were discovered by accident and were not ruptured. The symptoms in these cases were mainly from the pituitary adenoma [2]; the main manifestations were hormonal disorders and optic nerve compression symptoms. A small number of pituitary adenomas presented as hemorrhagic apoplexy. The main reason for apoplexy is the rupture of nutrient arteries inside the tumor [3, 4]. Among the cases of pituitary adenoma associated with aneurysm, hemorrhagic apoplexy caused by the associated aneurysm is rare. This article reports one case of pituitary adenoma apoplexy caused by rupture of an anterior communicating artery aneurysm and reviews the literature to increase the understanding of this disease.

## Case presentation

The patient was a 49-year-old male who came to our hospital due to “sudden severe headache accompanied by nausea and vomiting for three hours.” The patient had a medical history of a space-occupying lesion in the sellar region, which was regarded as a pituitary adenoma and had not been treated. The patient had no history of hypertension or diabetes mellitus. The examination of neurological system revealed the following: the patient was conscious and could answer questions correctly, the left eye vision was 0.2, the right eye vision was 0.5, there was bilateral temporal hemianopsia, and optic nerve atrophy was observed in the fundus. The patient had flexible limbs, normal muscle tension, grade V muscle strength, positive Kernig signs, and a grade III Hunt-Hess classification. The routine physical examination revealed that the skin was delicate, the pubic hair was sparse, and the breasts were developed. The laboratory tests showed that prolactin (PRL) was 1020 mIU/L (normal range, 70.81–566.46 mIU/L), growth hormone (GH) was 0.60 ng/mL (normal range, 1–5 ng/mL), and other indicators did not show any abnormalities.

**Fig. 1:**
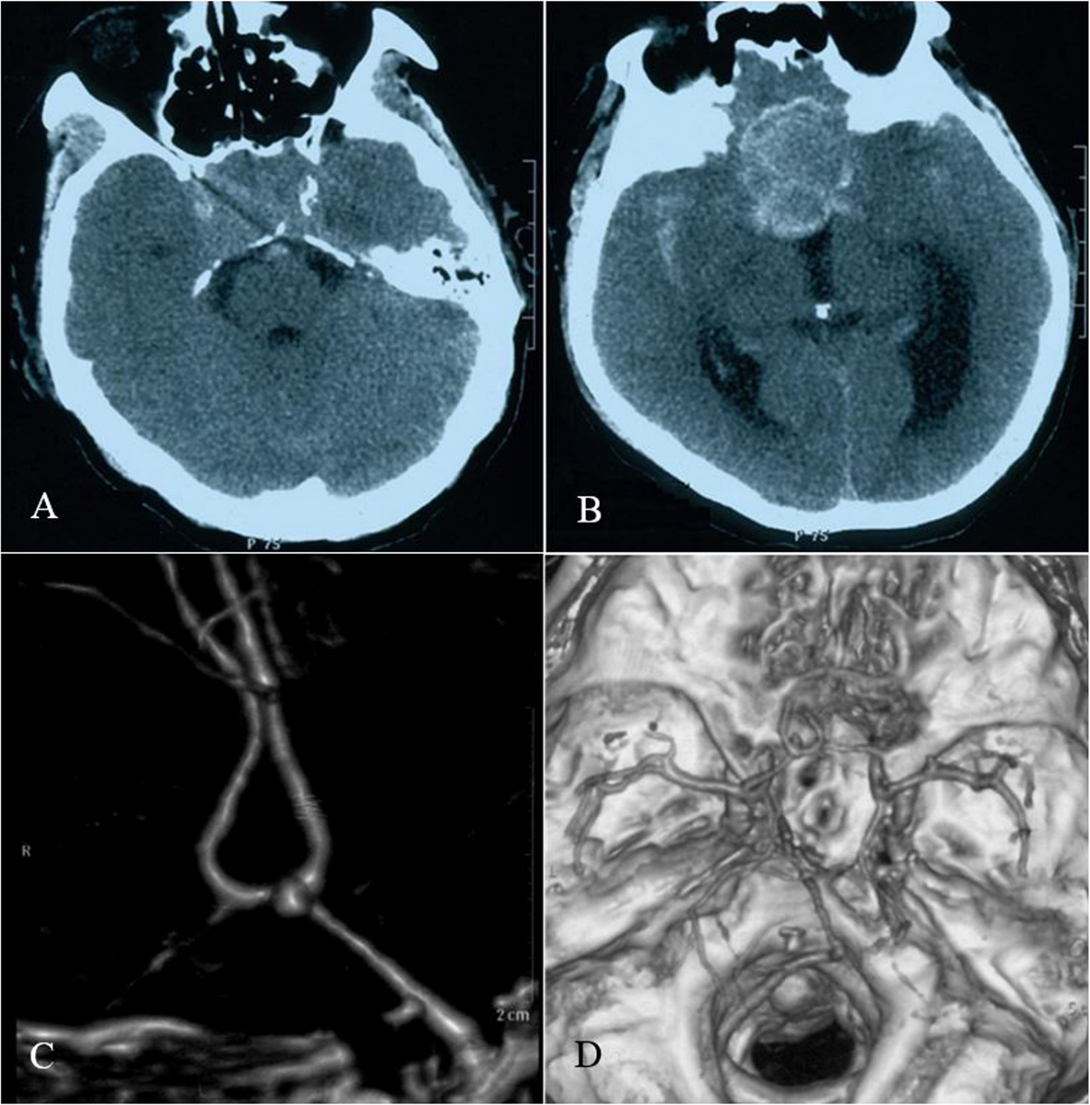
Cranial CT and CTA images. **a**, **b** Cranial CT showed space-occupying lesions in the sellar region and local bone destruction. At higher levels, the tumor image could be observed, and the boundary was clear. There appeared to be a capsule, the size of which was approximately 3 cm × 4 cm. The density inside and surrounding the tumor increased, patchy hemorrhage was observed, and a cord-like high-density image was observed in the bilateral sylvian cisterns. The findings were considered to be consistent with subarachnoid hemorrhage. **c**, **d** Cranial CTA revealed a cystic-like protuberance, identified as an aneurysm, in the anterior communicating artery. The neck of the aneurysm shifted toward the A1 segment of the left anterior cerebral artery. The left anterior cerebral artery was thicker. The Willis Circle was compressed and pushed to the lateral side

**Fig. 2:**
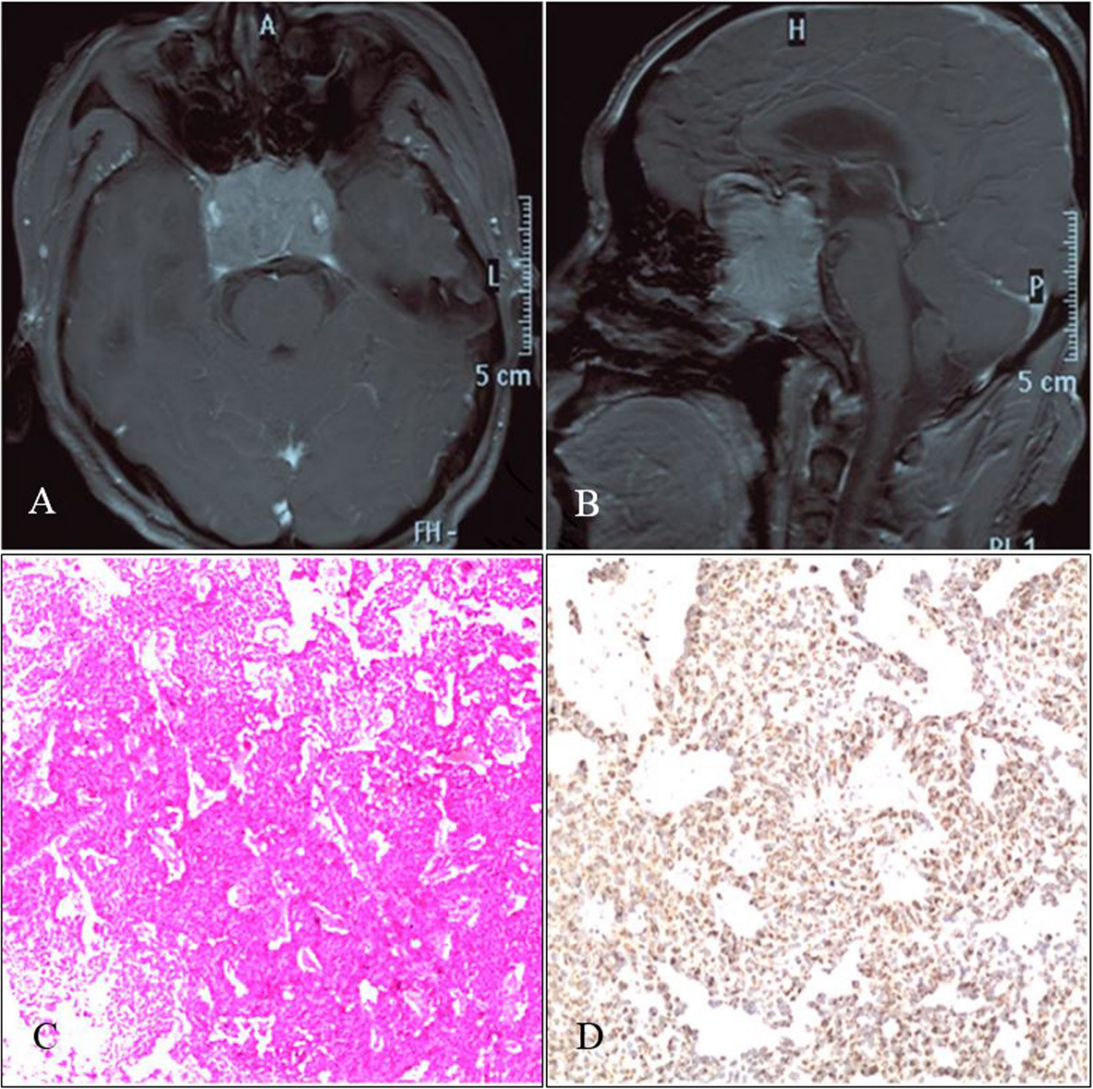
Cranial MRI imaging and pathological results. **a**, **b** The axial and sagittal planes of the cranial-enhanced MRI clearly showed the tumor in the sellar region. The tumor was significantly enhanced, the boundary was clear, the morphology was irregular, the bilateral carotid arteries were encased, and the size was approximately 5 cm × 4 cm × 3 cm. **c** HE staining (×200) revealed cells with a diffuse distribution. The morphology was consistent, there were fewer interstitial cells, and sinusoid capillaries were observed. **d** The immunohistochemistry results were PRL(+). Pathology confirmed that the lesion was a PRL-type pituitary adenoma

Emergency cranial computed tomography (CT) revealed space-occupying lesions in the sellar region with suprasellar expansion, local bone mass destruction, increased density in and around tumors, patchy hemorrhage, and high-density cord-like images in the bilateral sylvian cisterns. The findings were considered to indicate subarachnoid hemorrhage (Fig. 1a, b). Further cranial CT angiography (CTA) examination showed an anterior communicating artery aneurysm; the neck of the aneurysm was shifted toward the left anterior cerebral artery, and the Willis Circle was compressed and pushed to the lateral side (Fig. 1c, d). In addition, cranial magnetic resonance imaging (MRI) revealed that the tumor in the sellar region was significantly enhanced, the boundary was clear, the shape was irregular, and the bilateral carotid arteries were encased by the tumor; the tumor size was approximately 5 cm × 4 cm × 3 cm (Fig. 2a, b). Given the combination of the patient’s medical history, MRI scans, and laboratory examinations, the confirmed diagnosis of the patient was pituitary adenoma apoplexy and a ruptured anterior communicating artery aneurysm.

**Fig. 3:**
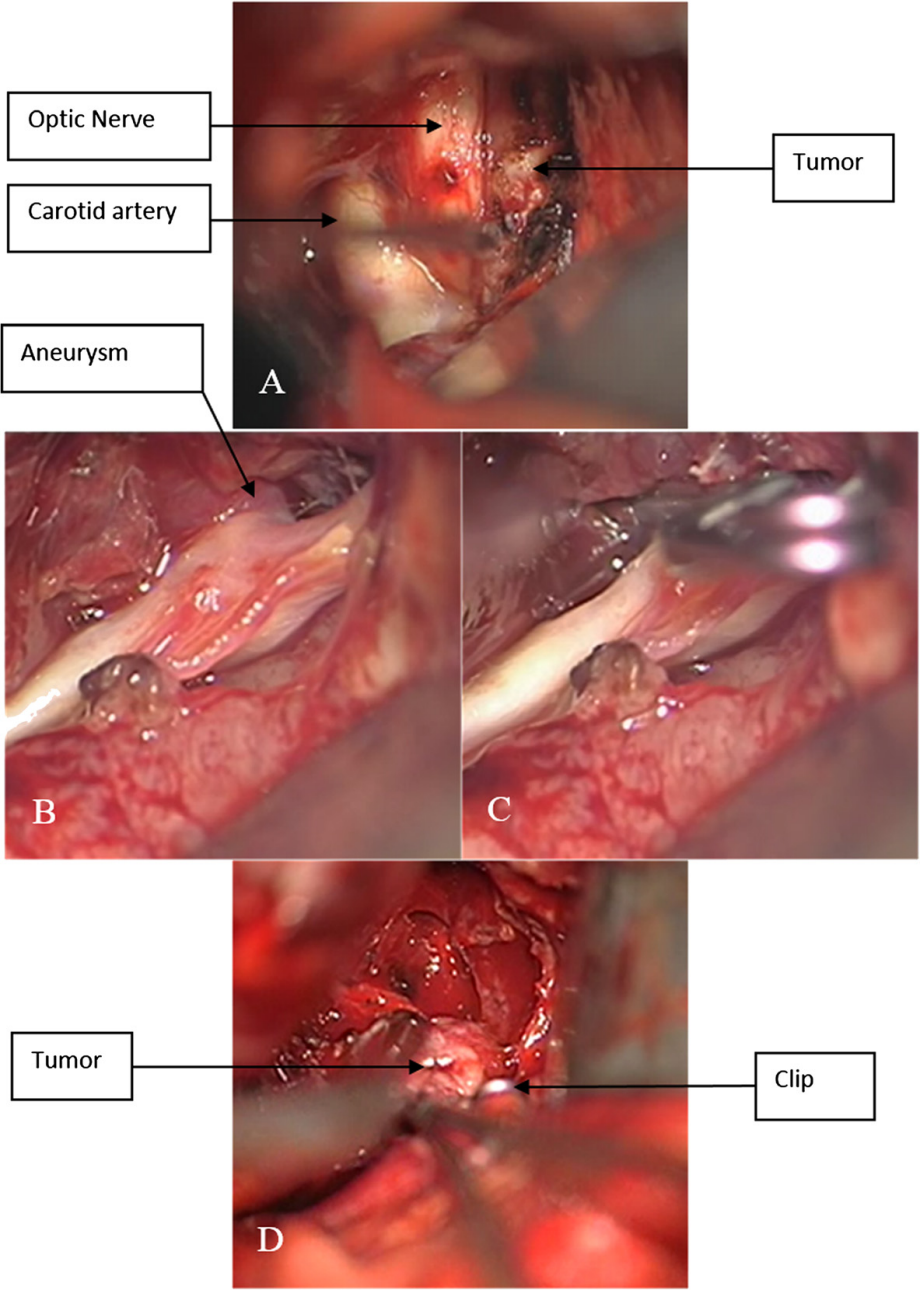
Craniotomy images. **a** A tumor in the optic chiasm was observed. The carotid artery, optic nerve, and tumor were clearly displayed. **b**, **c** Images of the anterior communicating artery aneurysm (before and after clipping) are displayed. **d** The surroundings were explored after aneurysmal closure. The aneurysm was observed to be encased by the surrounding tumor

**Fig. 4:**
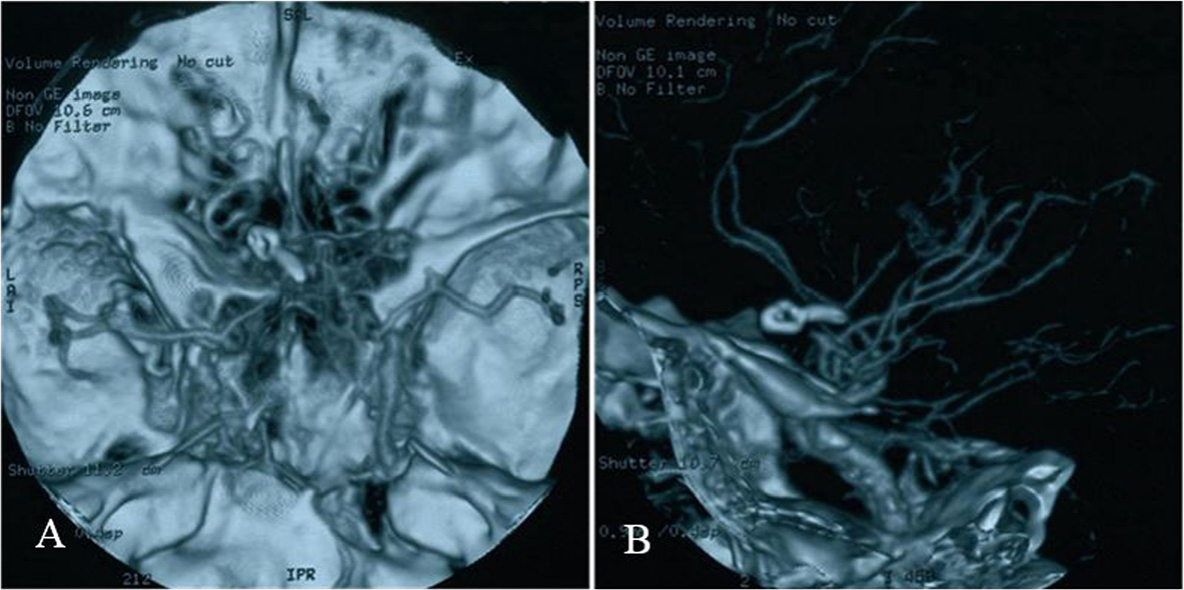
CTA after aneurysm clipping. **a**, **b** The image of the aneurysm clip is clearly displayed. The anterior communicating artery aneurysm was completely clipped and closured. There was no residual neck of the aneurysm. The morphology and course of the Willis Circle was normal, without compression and displacement changes

Anterior communicating artery aneurysm clipping and tumor resection were scheduled. The surgery used the left pterional approach. The tumor was observed in the optic chiasm space; after the tumor was partially resected, the sample was immediately sent for a pathological examination. The pathological hematoxylin-eosin (HE) stain demonstrated a diffuse distribution of cells; the shape was constant, there were few interstitia, and sinusoid capillaries were observed. The immunohistochemistry was PRL positive and confirmed the lesion to be a PRL-type pituitary adenoma (Fig. 2c, d). The anterior communicating artery aneurysm was observed after separation was conducted along the A1 segment of the anterior cerebral artery to the anterior communicating artery. A hematoma was observed in the surroundings, indicating the ruptured aneurysm. The aneurysm pointed to the contralateral side. After the neck of the aneurysm was exposed, the aneurysm was clipped, and the surroundings were explored. The aneurysm was found to be encased by the tumor. The tumor portion was resected (Fig. 3a–d). After the surgery, the patient recovered well, and his vision was significantly improved. Cranial CTA was conducted again after 1 week of surgery; the results showed that the aneurysm was well closured, there was no residual neck in the aneurysm, and the morphology and course of the Willis Circle were normal (Fig. 4a, b). The patient recovered well and was discharged. After discharge, the patient received gamma knife radiotherapy. After a half year of follow-up, the patient experienced no clinical complications.

### Discussion

Pituitary adenoma combined with intracranial aneurysm is not rare. The studies of Pant et al. demonstrated that approximately 7.4 % of pituitary adenomas were combined with an aneurysm; 97 % of these aneurysms were located in the anterior circulation [1]. Because both pituitary adenomas and cerebral aneurysms are common intracranial lesions, these two lesions sometimes coincide and occur concurrently; however, to date, no exact incidence of pituitary adenoma associated with intracranial aneurysm has been determined; nonetheless, there is a general consensus that intracranial aneurysms occur more frequently in association with pituitary adenomas than among the general patient population. In addition, associations with intracranial aneurysms are generally thought to be stronger for patients with pituitary adenomas than for patients with other brain tumors, although the manner in which a pituitary adenoma contributes to the formation of an intracranial aneurysm remains unclear [5]. Thus, in addition to speculation of an accidental encounter between a pituitary adenoma and an intracranial aneurysm, there exist other hypotheses to explain the manifestations in the described case.

Because the pituitary adenoma is a hormone-secreting tumor, the secreted hormones can induce aneurysm occurrence and growth. The study of Jakubowski et al. showed that in the cases of pituitary adenoma associated with an aneurysm, the pituitary adenomas were mostly of the GH type; by contrast, among the PRL-type pituitary adenomas that had the highest incidence, the probability of association with an aneurysm was very low. Therefore, it was speculated that although GH might induce aneurysm development and growth, PRL may have only weak capabilities in this regard [1, 6]. Despite these weak capabilities, among 5 cases of pituitary adenoma apoplexy or subarachnoid hemorrhage that we reviewed, at least 4 of the cases involved PRL-type adenomas (Table 1). Oh et al. examined 800 cases of pituitary adenoma associated with aneurysm and demonstrated that older age and the existence of a cavernous sinus invasion were correlated with an increased incidence of intracranial aneurysm among patients with pituitary adenomas [5]. Numerous hypotheses to explain this phenomenon have been proposed, including a direct mechanical effect of the pituitary adenoma on the vasculature, direct infiltration by the tumor, and GH production leading to arteriosclerotic and degenerative changes in the arterial walls of the circle of Willis, thereby, predisposing patients to the formation of aneurysms [7–9]. Therefore, the underlying reasons for the association between pituitary adenomas and aneurysms require continued exploration and study.

**Table 1:** Patients with pituitary adenoma and ruptured aneurysm presenting with pituitary apoplexy or intracranial hemorrhage

Patient no.	Author/year	Age (years)/sex	Clinical manifestations	CT imaging	Aneurysm location	Pathological type	Treatment	Outcome
1	Suzuki/2001 [11]	46/M	Headache, vomiting, and visual impairment	Pituitary tumor with intrasellar and parasellar hemorrhage	Right intracavernous carotid artery	Prolactinoma	Transsphenoidal surgery; endovascular occlusion of the right internal carotid artery by platinum coils	Good
2	Okawara/2007 [12]	48/F	Headache and visual impairment	Pituitary tumor with intratumoral hemorrhage, intraventricular hemorrhage and subdural hemorrhage	Left intracavernous carotid artery	Prolactinoma	Aneurysm coiling by platinum coils; administration of terguride to shrink the pituitary adenoma	Good
3	Song/2014 [13]	31/M	Headache, vomiting and blurred vision	Subarachnoid hemorrhage in basal cisterns	Posterior communicating artery	Pituitary adenoma with hemorrhagic necrosis	Aneurysm clipping; during surgery, pituitary apoplexy was discovered and removed	Good
4	Almeida/2014 [15]	53/M	Headache accompanied by mental confusion	Subarachnoid hemorrhage, interhemispheric hematoma, and suprasellar expansive lesion	Anterior communicating artery	Prolactinoma	Aneurysm clipping; partial resection of the pituitary tumor	Good
5	Present case	49/M	Sudden headache accompanied by a pituitary adenoma that had been untreated for three years	Pituitary adenoma hemorrhage and significant subarachnoid hemorrhage	Anterior communicating artery	Prolactinoma	Aneurysm clipping; major resection of the pituitary tumor	Good

Among the pituitary adenomas associated with aneurysms, aneurysms at the cavernous sinus segment are the most common. This type of aneurysm is mostly embedded inside the pituitary adenoma below the sellar diaphragm [10]. In cases involving pituitary adenoma combined with an aneurysm, most of the aneurysms are inactive and unruptured. The clinical symptoms are mainly caused by hormones secreted by pituitary adenomas or by compression on the surrounding brain tissues and optic nerve [3]. However, pituitary adenomas have a rare hemorrhagic clinical presentation, namely, tumor apoplexy; thus, the combined aneurysm factor should be considered. A simple pituitary apoplexy is mainly caused by the rupture and hemorrhage of blood-supplying arteries within tumors; the major presentations are a hemorrhage confined within the tumor, a rapid increase in the tumor volume caused by this hemorrhage, and severe headache and vision loss caused by compression of the optic nerve and surrounding dura [4]. When a pituitary adenoma is combined with an aneurysm under the sellar diaphragm, if the aneurysm is ruptured, it may also cause the typical pituitary adenoma apoplexy symptoms. For example, in 2001, Suzuki et al. reported one case of an intracavernous carotid artery aneurysm embedded in a pituitary adenoma. The hemorrhage after rupture was confined inside the tumor, and the presentation included the typical pituitary adenoma apoplexy symptoms and imaging results [11].

At times, hemorrhage caused by aneurysm rupture is not limited to the tumor; for instance, in 2007, Okawara et al. reported a case (similar to the case described in this report) that involved an intracavernous carotid artery aneurysm located in a pituitary adenoma. The manifestations of the hemorrhage that occurred after aneurysm rupture included intratumoral hemorrhage, intraventricular hemorrhage, subdural hemorrhage, pituitary adenoma apoplexy, and other hemorrhages [12]. In addition to aneurysms in the cavernous sinus segment, other aneurysms can also cause pituitary adenoma apoplexy; for instance, in 2014, Song et al. reported a case in which a posterior communicating aneurysm caused pituitary adenoma apoplexy and in which invasive growth had caused the pituitary adenoma to wrap around the aneurysm [13].

When the aneurysm that is combined with a pituitary adenoma is not inside the cavernous sinus, the aneurysm may be wrapped by the invasive pituitary adenoma that grows through the sellar diaphragm. Once rupture and hemorrhage occur, the aneurysm may not be confined inside the tumor and may enter into the subarachnoid space to combine with subarachnoid hemorrhage. The presentation is pituitary adenoma apoplexy combined with subarachnoid hemorrhage. This disease is very rare. The case reported in this article falls into this category. The rupture of the anterior communicating artery aneurysm caused hemorrhage inside the pituitary adenoma and extensive subarachnoid hemorrhage. During the surgery, the aneurysm was confirmed to be ruptured; there were blood clots in the surroundings, and the aneurysm was inside the pituitary adenoma. The patient successfully underwent tumor removal and aneurysm clipping. Because of his ignorance of medicine and his difficult living conditions, the patient did not accept a dopamine agonist to treat his prolactinoma; instead, he chose to adopt a wait-and-see approach to his disease even though he lost a portion of his eyesight. If the prolactinoma had been dissolved with medication, the treatment of the aneurysm would have been less complex.

A case involving mechanisms similar to those of the case discussed in this article was reported in 2006 by Shahlaie et al., who described how the rupture of an anterior communicating artery aneurysm caused pituitary gland apoplexy and subarachnoid hemorrhage [14]. Because the anterior communicating artery is higher, not all pituitary adenomas can grow upward to encase it. For example, in 2014, Almeida et al. reported a case of pituitary adenoma associated with an anterior communicating artery aneurysm and demonstrated that a hemorrhage after aneurysm rupture could not enter the pituitary adenoma due to the elevated location of the aneurysm; therefore, the only observed presentations of this hemorrhage were an interhemispheric hematoma and a subarachnoid hemorrhage [15]. Overall, in pituitary adenoma apoplexy combined with aneurysm rupture, based on the aneurysm’s location and relationship with the sellar diaphragm, the presentation may (or may not) be combined with subarachnoid hemorrhage.

Therefore, when hemorrhagic apoplexy occurs in a pituitary adenoma and is combined with subarachnoid hemorrhage, the possibility of an associated aneurysm should be considered. However, the literature reports that simple pituitary adenoma apoplexy might also present subarachnoid hemorrhage. For example, Nakahara et al. reported one case showing that subarachnoid hemorrhage occurred when the pituitary adenoma was not associated with an aneurysm. This condition was considered to result from arterial laceration caused by stretching of the adjacent arteries (such as the superior hypophyseal artery) by tumor expansion and invasive growth [16]. Currently, transsphenoidal resection is the main treatment method for pituitary adenomas [17]. If the aneurysm diagnosis is missed before surgery, surgical resection of the tumor can easily cause aneurysm rupture and hemorrhage; the tumor-loading artery cannot be found in the narrow and small surgical space to control the bleeding, thus, causing disastrous consequences. However, for pituitary adenoma apoplexies caused by intracranial aneurysms, if the aneurysms could be found, aneurysm coiling combined with transsphenoidal pituitary adenoma resection remains a good treatment option [11, 12].

## Conclusions

Therefore, the purpose of this article is to remind physicians to consider the possibility of combined aneurysm rupture when a pituitary adenoma causes apoplexy combined with subarachnoid hemorrhage. When the lesion is combined with an aneurysm, supratentorial craniotomy should be selected if possible. During aneurysm clipping, the pituitary adenoma can also be resected, thus, preventing the aneurysm from rupturing again during transsphenoidal surgery. Aneurysm coiling combined with transsphenoidal pituitary adenoma resection may be a treatment option for such cases.

## Consent

Written informed consent regarding the publication of this case report and its accompanying images was obtained from the patient. Copies of the written consent form are available for review upon request.
